# Radiotherapy for Non-Hodgkin’s lymphoma: still standard practice and not an outdated treatment option

**DOI:** 10.1186/s13014-016-0690-y

**Published:** 2016-08-30

**Authors:** Michel Zimmermann, Christoph Oehler, Ulrich Mey, Pirus Ghadjar, Daniel Rudolf Zwahlen

**Affiliations:** 1 Department of Radiation Oncology, Kantonsspital Graubünden, Loëstrasse 170, 7000 Chur, Switzerland; 2 Department of Medical Oncology and Haematology, Kantonsspital Graubünden, Chur, Switzerland; 3 Department of Radiation Oncology, Charité Universitätsmedizin Berlin, Berlin, Germany

## Abstract

Two large, recently published observational studies demonstate a clear down-trend in the use of radiotherapy (RT) over the last 15 years, both in the setting of follicular and diffuse large B-cell lymphoma. This change of practice might have a negative impact on clinical outcome. Even within the context of modern systemic therapy, omission of RT translates not only into a shorter progression-free survival (PFS), but also into a worse overall survival (OS). RT should therefore remain standard practice.

This short review is aiming to summarize current guidelines and the best evidence available in the management of non-Hodgkin’s lymphoma. Potentially practice changing, ongoing trials will be highlighted.

## Introduction

In the management of non-Hodgkin’s lymphoma (NHL), radiotherapy (RT) has been the first treatment leading to long-lasting remission and even cure for a number of patients at the beginning of the 20^th^ century [1, 2]. More recently, combination chemotherapy and immuno-chemotherapy with the addition of rituximab has evolved with increasing efficacy and now plays a major role in the management of many B-cell NHLs [3].

RT is a local treatment, and only patients with early stage disease can be treated with curative intent using RT as a single modality. RT is currently used as the primary treatment in early stage, indolent lymphomas. For localized aggressive lymphomas, RT is often delivered as consolidation therapy after systemic chemotherapy in a multimodality therapy approach. For patients with advanced NHL, indications for the use of RT are less obvious and the evidence less robust [4].

A decreasing use of consolidative RT after multiagent chemotherapy has been observed over the last 15 years. In a recent large retrospective study, two thirds of patients with early stage diffuse large B-cell lymphoma (DLBCL) are now treated with (immuno-) chemotherapy alone; the authors observed a decline in the use of consolidation RT from a peak of 47 % in 2000 to a nadir of 32 % in 2012 (*p* < 0.001) [5]. Omission of radiotherapy and abandonment of combined-modality approaches in current clinical practice might not always be fully evidence-based, and prompted us to perform a short review of the literature.

We conducted a search of MEDLINE using PubMed in January 2016, dated back to January 1, 2004. The MeSH search terms included “low-grade lymphoma” and “high-grade lymphoma” and used the limiting terms “radiation therapy”, “randomized controlled trials”, and English language. We further focused our search by excluding mantle-cell, marginal zone lymphoma and other rare types of lymphomas. We also reviewed reference lists of current guidelines; precedence was given to the National Comprehensive Cancer Network (NCCN) recommendations and European Society for Medical Oncology (ESMO) Clinical Practice Guidelines [6–8].

### Follicular lymphoma (FL)

FL represents the most common indolent NHL in the USA and Europe, accounting for an estimated 20-30 % of all NHL [9]. The genetic hallmark of FL, the translocation t(14;18)(q32;q21), results in the constitutive overexpression of the bcl 2 protein, impairing the normal germinal centre apoptotic programme [10].

Initial workup should include an adequate staging (including bone marrow examination) to ensure precise definition of the extent of the disease. Recently, the use of PET imaging - in clinical trials and clinical practice - has been recommended as the preferred option for staging and remission assessment of all FDG-avid NHLs, including follicular lymphoma by an International Consensus group [11]. In FL, the use of positron emission tomography (PET) scanning is particularly useful in patients presenting with early stage disease to confirm localized disease before initiating RT [7].

RT remains the current standard of care for patients with low-grade (grade 1-2), early stage FL (non-bulky stage I-II), which represent 10-15 % of all FL [7]. Other options in selected cases include observation, immunotherapy (rituximab) and chemotherapy. FL grade 3b is regarded as an aggressive lymphoma and treated like DLBCL.

Despite NCCN and ESMO guidelines endorsing RT as the preferred initial management [6, 7], RT use in patients with early-stage low-grade FL continues to decline. In the largest retrospective cohort study reported so far (35,961 patients), use of RT dropped from 37 % in 1999 to 24 % in 2012 (*p* < 0.0001), correlating with an increase of observation from 34 % to 44 % (*p* < 0.0001) over the same period [12]. In this paper, the use of RT was associated with a significant 12 % absolute improvement in the 5-year and a 14 % absolute improvement in the10-year overal survival (OS) rate. An identical 14 % absolute 10-year OS benefit in patients receiving RT was noted in an older population-based analysis from 1973 through 2004 from the SEER data set [13]. These data suggest that even within the context of modern systemic therapy, RT significantly contributes to survival for patients with early-stage low-grade FL and should remain standard practice.

These results have been challenged by a retrospective multicenter observational study (National LymphoCare Study). In this series, only 111 out of 474 patients (23.4 %) with stage I FL received RT as single modality treatment [14]. Excellent outcomes with different treatment approaches were achieved, suggestive of a potential PFS benefit for systemic therapy plus RT or systemic therapy plus rituximab over RT alone, but without any OS difference [15]. However, several flaws make it difficult to draw any conclusions on treatment choices from this retrospective analysis. The National LymphoCare study was not designed to compare different therapeutic options in early-stage FL, and selection of treatment was not based on predefined entry criteria [10, 16]. Furthermore, a potential conflict of interest might arise from the partial funding of this study by pharmaceutic companies (Genentech and Biogen).

The dogma of early-stage, indolent lymphoma as a pure localized disease (amenable to cure with RT) has to be challenged, as nearly half of patients with early-stage FL will relapse within ten years, almost exclusively in distant non-irradiated areas [17]. It should be noted that with the increasing integration of PET-CT into initial management, improved staging accuracy may translate into improved disease control and survival rates for early-stage FL, by excluding patients with occult, distant disease [18]. For the latter, lymphoma remains the leading cause of death [19].

In this context, rituximab represents an ideal systemic drug with a low toxicity profile, aiming to eliminate circulating lymphoma cells as well as distant subclinical involvement, and would complement nicely RT, which targets macroscopic disease only. Furthermore, rituximab proved as a radiosensitizer in vitro, enhancing radiation-induced apoptosis and cell growth delay [20]. Prospective studies are therefore urgently needed, to answer the question if single-agent rituximab, rituximab combined with involved-field radiotherapy (IFRT) (sequential and/or concomitant), or even radioimmunotherapy offers any advantage over IFRT alone [21]. So far, we are only aware of a single prospective, multicentric phase II-study (MIR trial, 85 patients) in the field. The treatment included a first sequence of rituximab (4 weekly cycles; 375 mg/m^2^ body surface), a four week treatment gap with a restaging CT / planning CT of the involved region in week seven, and followed by another block of rituximab (4 weekly cycles) concurrently with an IFRT of 40 Gy for macroscopic tumor or 30 Gy in case of a complete remission after rituximab induction [22]. Preliminary results were presented in abstract form in 2012; the authors stated that this combined treatment was well tolerated; 2-year PFS was similar to historical data with large field RT without the accompanying toxicity and was superior to historical data of IFRT only [23]. Mature results are pending, however.

In current practice, we consider that RT should not be omitted outside clinical trials. For patients with low-grade, early-stage FL, general dose guidelines are 24-30 Gy and 24-36 Gy according to NCCN and ESMO recommendations [6, 7].

The British National Lymphoma Investigation (BNLI) randomized study showed that 24 Gy in 12 fractions was as effective for local control as 40 Gy in 20 fractions in terms of overall response and within field progression [24]. This attractive 24 Gy dose recommendation has not been universally implemented so far, as the trial presents some shortcomings, including a greater heterogeneity both in terms of histological diagnosis (FL grade 1-2 making only 59 % of the 289 patients with indolent lymphoma) and in the evaluation of treatment response (no consequent 3D imaging during follow-up).

Very low-dose radiotherapy (4 Gy) has been evaluated in a Dutch phase II study of 109 patients with recurrent indolent lymphoma (98 cases with FL), with an overall response rate of 92 %. The subgroup of 67 patients with complete response (CR) showed a median time to progression of 25 months and a median time to local progression of 42 months [25].

To address prospectively the issue of very low-dose RT, the FORT trial randomized 614 sites (in 548 patients) to receive either 24 Gy or 4 Gy. Results clearly showed that 4 Gy was inferior to 24 Gy in terms of time to local progression (HR of 3.4; 95 % CI: 2.09-5.55; *p* < 0.001) [26]. The FORT trial has been criticized not only for presenting similar flares to the previous dosage study (40 Gy vs 24 Gy) as mentioned above, but also for not stratifying patients and assessing local response according to the initial size of lesions; the non-inferiority of the 4 Gy regimen might not be excluded for smaller FL lesions [27]. To illustrate their point further, the same investigators highlight the encouraging results achieved with orbital lymphoma, where the 2x2 Gy schedule appears to be quite a valuable option, with two retrospective studies reporting a local PFS of 100 % at 2 years [28, 29].

Taken together, 24 Gy can be considered as the lowest standard dose for curative treatment of most patients with low grade, early-stage FL, until a lower dose (that is in between 4 Gy and 24 Gy) is explored properly [16].

For patients with asymptomatic, low-tumor burden stage III-IV disease, FL is considered as a chronic disease; a “watch and wait” strategy is usually recommended. The natural course of disease is characterized by spontaneous regression in 10-20 % of cases [7]. Systemic treatment (rituximab ± chemotherapy) should only be initiated when a patient presents with indications for treatment (Groupe d’Etude des Lymphomes Folliculaires (GELF) criteria) [6]. Future research protocols will also integrate second-generation anti-CD20 antibodies (ofatumumab and obinutuzumab), lenalidomide, mTOR inhibitors (temsirolimus and everolimus) Bcl-2 or Bruton’s tyrosine kinase (BTK) inhibitors.

Local palliation with low-dose RT (2x2 Gy) may be used in selected cases. This treatment regimen provides effective symptomatic relief for tumor bulk of all sizes, with an overall response rate of 81 % [30]. Should the patient progress again locally without significant systemic progression of disease, local treatment with 2x2 Gy can be repeated. This low-dose RT regimen remains an excellent option in the palliative and relapse setting, especially for patients with poor performance status, and should be practiced more frequently [16].

### Diffuse large B-cell lymphoma (DLBCL)

The most common type of aggressive B cell lymphoma is DLBCL, accounting for approximately 30 % of NHLs diagnosed annually [31]. Gene expression profiling studies have revealed significant heterogeneity within DLBCL. Incoroporation of this information into treatment algorithms awaits further investigation [6].

#### Early-stage DLBCL

Prognosis is very favorable for patients with early-stage disease with no adverse risk factors (i.e. elevated LDH, bulky disease, older than 60 years or ECOG performance status of 2 or more).

For patients with non-bulky (<7.5 cm) early-stage disease (stage I-II), R-CHOP (3 cycles) + RT, or R-CHOP (6 cycles) +/- RT is recommended by the NCCN guidelines [6]. The ESMO guidelines recommend 6 x R-CHOP for patients ≤ 60 years of age and 6 x R-CHOP-21 or 6 x R-CHOP-14 plus 2 x R for patients 60-80 years of age, respectively [8]. Patients with bulky disease (7.5 cm or larger) may be more effectively treated with 6 cycles R-CHOP + consolidative IFRT. These recommendations are mainly based on SWOG 8736 [32, 33] and the MabThera International (MInT) trial [34, 35].

When indicated, consolidative RT should be delivered up to a total dose of 30-36 Gy for patients achieving CR after chemotherapy plus rituximab, while patients achieving only partial response (PR) after induction therapy should receive 40-50 Gy according to NCCN recommendations; ESMO doesn’t provide any general dose guidelines for RT [6, 8].

The role of rituximab has been evaluated in the MInT trial, a randomized phase 3 trial comparing 6 cycles of CHOP-like chemotherapy to 6 cycles of CHOP-like chemotherapy plus rituximab. Consolidation RT was included for all extranodal sites of disease or any site greater than 7.5 cm. The trial found a benefit for rituximab-containing immunochemotherapy with a 6-year PFS rate of 80.2 % vs 63.9 % (*p* < 0.001) and a 6-year OS rate of 90 % vs 80 % (*p* = 0.004).

In the pre-rituximab era, the role for consolidative RT after chemotherapy was examined in four landmark randomized trials: SWOG 8736, GELA LNH 93-1, ECOG 1484, and GELA LNH 93-4.

In the SWOG 8736 trial, 3 cycles of CHOP followed by IF-RT were superior to 8 cycles of CHOP alone, both for PFS (5-year estimated PFS 77 % vs. 64 %; *p* = 0.03) and OS (5-year estimated OS 82 % vs 72 %; *p* = 0.02). However, 10-year follow-up suggested an increase in late recurrence in patients assigned combined treatment [33]. Extended survival data with more than 17 years of follow-up showed similar outcomes, with continuous treatment failure and without a PFS plateau in either arm [36].

The Groupe d’Etude des Lymphomes de l’Adulte (GELA) LNH 93-1 evaluated the chemotherapy regimen ACVBP alone, compared with 3 cycles of CHOP and IFRT in young patients (≤60 years). The ACVBP regimen was associated with a better 5-year event-free survival (EFS) (82 % vs 74 %, *p* = 0.001) and 5-year OS (90 % vs 81 %, *p* = 0.001) than abbreviated chemotherapy and IFRT [37]. The intensive chemotherapy ACVBP regimen has not been widely adopted, owing to concerns about acute and late toxicities [38].

The Eastern Cooperative Oncology Group (ECOG) administered 8 cycles of CHOP to patients with stage I-II diffuse aggressive lymphoma, and randomized those achieving complete response by CT (172 patients) between observation and 30 Gy IFRT. The 6-year disease-free survival (DFS) was 73 % for low-dose RT versus 56 % for observation (*p* = 0.05), with no improvement in OS (87 % vs 73 %, *p* = 0.24) [39].

The GELA LNH 93-4 trial included elderly patients (>60 years) with stage I-II aggressive lymphoma. Patients were randomly assigned between 4 cycles of CHOP alone, or followed with IFRT. The 5-year estimates of EFS (64 % vs 61 %, *p* = 0.6) and OS (68 % vs 72 %, *p* = 0.5) did not differ between those receiving combined modality therapy and chemotherapy alone [40].

Taken together, these prospective trials from the pre-rituximab era suggest that IFRT might improve local control for patients with early-stage DLBCL, without providing an OS advantage. It is likely that as systemic therapy improves, the impact of IFRT may be further diminished [38]. Still, complete omission of consolidative RT might be detrimental for some patients.

Using the National Cancer Data Base, Vargo et al. identified 59,255 patients with stages I and II DLBCL treated with multiagent chemotherapy alone or chemotherapy plus consolidative RT between 1998 and 2012 [5]. Median follow-up time was 60 months (interquartile range: 33-93). Use of combined-modality therapy significantly decreased from a peak of 47 % in 2000 to a nadir of 32 % in 2012 (*p* < 0.001). Estimated 5-year and 10-year OS rates were 75 % and 55 % for patients receiving chemotherapy alone, and 82 % and 64 % for patients receiving combined modality therapy (*p* < 0.001). In this large population-based retrospective trial, patients who received combined multiagent chemotherapy plus RT had a significant survival advantage over those treated with multiagent chemotherapy alone.

Ongoing clinical research is necessary to further establish the role of combined-modality therapy in early-stage DLBCL in the context of modern systemic therapies. A risk-adapted treatment approach might emerge, with the identification of “PET-negative” patients after initial rituximab-chemotherapy, who could be spared consolidation RT and attendant toxicities [36].

#### Advanced stage DLBCL

For patients with advanced stage disease (stage III-IV), treatment with 6-8 cycles of R-CHOP or more intensive treatment regimens like R-CHOEP, R-ACVBP or even treatment regimens including high-dose chemotherapy are recommended [8]. In selected cases, consolidation RT to bulky sites may be beneficial. These recommendations are mainly based on the GELA study (LNH 98-5), which was performed with elderly patients (60-80 years) with advanced DLBCL, randomized between 8 cycles of R-CHOP versus CHOP. At a median follow-up of ten years, PFS (36.5 % vs 20 %) and OS (43.5 % vs 28 %) were significantly in favor of R-CHOP [41].

Two randomized trials reported data comparing R-CHOP-21 with dose-dense R-CHOP-14 [42, 43]. These studies did not show an improvement in outcome for the dose-dense therapy over the R-CHOP 21 regimen [6]. Still, the R-CHOP-14 regimen is commonly used for the treatment of elderly patients outside clinical trials, based on the excellent results of the RICOVER-60 trial [44], the equal toxicity of both regimens, and the considerably shortened 12-week treatment with 6 R-CHOP-14 instead of the 24-week treatment with 8 R-CHOP-21.

Assessment of response is best performed with PET scan at the end of treatment. Interim PET scans can produce false positive results. In a prospective study evaluating the significance of interim PET scans after 4 cycles of R-CHOP-14, only five out of 38 patients with a positive interim PET scan had a biopsy demonstating persistent disease. No difference in PFS outcome was noticed between interim PET-positive, biopsy negative patients, and interim PET-negative patients [45]. Interim PET scan was also shown to be only of limited prognostic value in a larger prospective, international single-arm study (SAKK 38/07), which investigated the prognostic value of PET scan after two cycles of dose-dense R-CHOP-14 (out of 6 cycles followed by two cycles of rituximab) for previously untreated DLBCL patients. Patients with a negative interim PET scan achieved a complete response in 100 % of cases at the end of treatment, compared with 71 % for PET-positive patients. While the two-year event-free survival (EFS) was significantly shorter for PET-positive compared with PET-negative patients (48 % v 74 %; *p* = 0.004), OS at 2 years was not statistically significant, by both local and central review of the PET results [46].

Although interim PET scan is not recommended routinely, the end-of-treatment PET scan is highly predictive of PFS. Among 88 first-line DLBCL patients treated with 6-8 R-CHOP courses, patients with a negative final PET scan achieved a 2-year PFS of 83 % vs. 64 % for patients with a final positive PET scan (*p* < 0.001) [47].

Once the planned course of R-CHOP to a total of 6-8 cycles is completed, an end-of-treatment PET scan will be performed 6-8 weeks later. For patients achieving PET-negative CR, observation is an option with strong consideration of consolidative RT to initially bulky disease or skeletal involvement [48]. For patients with PR (after completion of initial therapy), a rescue treatment with high-dose chemotherapy and autologous stem cell transplantation (ASCT) might be an option in selected cases.

The German High-Grade Non-Hodgkin’s Lymphoma Study Group is currently running a prospective, phase III study for aggressive B-cell lymphoma (UNFOLDER21/14 trial; NCT00278408). Patients are initially randomized to receive either R-CHOP 21 or R-CHOP-14, with a second randomization to RT or observation for patients with extranodal or bulky disease (2 x 2 factorial design). The two arms without RT to bulky (defined as ≥ 7.5 cm) or extralymphatic sites were closed because of significant inferiority with respect to EFS on the second planned interim analysis with 285 patients, suggesting a benefit of RT in patients with bulky disease [49]. Final results are awaited, as they will allow to better define the place of consolidation RT in the setting of aggressive DLBCL, in the modern era of rituximab and advanced RT treatment delivery techniques.

For elderly patients (>60 years of age), an amendment to the RICOVER-60 trial (RICOVER-noRTh) allowed to prospectively compare two cohorts of patients with DLBCL [50]. Both cohorts were treated with 6 x R-CHOP–14 + 2R (R-CHOP administered every 2 weeks plus two additional applications of rituximab) before receiving either IFRT (36 Gy) or no RT. A multi-variable per-protocol analysis limited to patients with bulky disease (i.e. excluding patients receiving unplanned RT) revealed HRs of 2.7 for EFS (*p* = 0.011), 4.4 for PFS (*p* = 0.001), and 4.3 for OS (*p* = 0.002) for patients not receiving consolidative RT. Even if patients were not randomly allocated to RT, this study provides strong support for adding RT to sites of bulky disease for elderly patients (>60 years of age) with aggressive B-cell lymphoma. As PET imaging was not part of this trial, it is not known whether RT can be omitted in patients with initial bulky disease who have a negative PET scan after completion of immunochemotherapy.

In a retrospective analysis of nine consecutive prospective trials from the German High-Grade Non-Hodgkin lymphoma Study Group, data from 3840 patients with newly diagnosed DLBCL were available. The study focused on the 292 patients (7.6 %) with initial skeletal involvement. Chemotherapy was delivered in combination with rituximab in 61 cases (20.9 %) and without rituximab in 231 cases (79.1 %). The analysis of the effect of RT was restricted to the 161 patients achieving PR or CR at the end of (immuno-) chemotherapy, out of studies where consolidation RT was allowed (mostly 36 Gy in 1.8-2 Gy per fraction). The 133 patients who received RT to sites of skeletal involvement had a significantly better 3-year EFS (75 % vs 36 %; *p* < 0.001) than the 28 patients not receiving RT, with a trend towards a better 3-year OS (86 % vs 71 %; *p* = 0.064). These data suggest a benefitial effect of RT in DLBCL patients with skeletal involvement [51].

For patients with non-CR after R-CHOP treatment, RT might be viewed more as a salvage treatment than pure consolidation after chemotherapy. We are currently mainly left with a large, retrospective study from 974 patients with advanced DLBCL (treated between 1980 and 1999) pooled from four different EORTC studies [52]. Out of these 974 patients, 227 achieved PR after eight cycles of doxorubicin-based chemotherapy. Salvage treatment strategy included IFRT (114 patients), second-line chemotherapy (93 patients), autologous stem cell transplantation (ASCT) or surgery (16 and 4 patients each). In multivariate analysis, RT was clearly the most significant factor affecting both OS and PFS. This study stems unfortunately from the pre-rituximab and pre-PET scan era, precluding the implementation of its conclusions in current management stategy.

In patients with advanced aggressive B cell lymphomas and partial metabolic response after R-CHOP treatment, the role of RT to residual disease has to be adressed in randomized trials, like the currently recruiting OPTIMAL > 60 trial for elderly patients (NCT01478542).

### Primary mediastinal large B-cell lymphoma (PMBCL)

PMBCL is relatively uncommon, comprising around 3 % of all NHL, and up to 10 % of DLBCL. It most often presents in young adults, with a median age of 35 [53]. Gene expression profiling is distinct from other types of DLBCL [54]. In common with nodular sclerosing Hodgkin lymphoma (HL) in the mediastinum, it is thought to originate from transformed thymic B-cells, and there is an intermediate entity which lies between these two types, currently termed mediastinal gray-zone lymphoma [55].

In the absence of randomized trials, optimal first-line treatment for patients with PBMCL is more controversial than other types of NHL, especially in the rituximab era [6]. The recently completed CALGB study (NCT00118209) comparing R-CHOP versus DA-R-EPOCH will provide useful information wether any particular chemotherapy regimen is superior.

The use of consolidation RT for PMBCL has been an historical standard of care, based upon poor results following chemotherapy alone prior to rituximab; furthermore the very poor outcomes for patients who develop recurrent disease highlighted the need to maximize cures at first attempt [56]. Considering the potential long-term toxicities of mediastinal RT, some investigators have elected to omit consolidation RT [56, 57]. The ongoing IELSG-37 study (NCT01599559) is currently evaluating the important question whether RT can be omitted in PMBCL patients, who have become “PET-negative” at the end of initial rituximab-chemotherapy regimens.

### Primary CNS lymphoma (PCNSL)

PCNSL accounts for approximatively 3 % of all primary CNS tumors. It is an aggressive form of NHL that develops within the brain, spinal cord, eye, or leptomeninges, without evidence of systemic involvement [6]. Ninety percent of non-HIV-associated PCNSL cases are of the diffuse large B-cell type, characterized by lymphoid clustering around small cerebral vessels [58]. The brain parenchyma is involved in more than 90 % of cases. Leptomeningeal involvement may occur in up to 30 % of patients. Ocular involvement may develop independently in ten to 20 % of patients.

The CHOP regimen induces responses of brief duration in patients with primary CNS lymphoma. This inefficacy is probably because the metabolite of cyclophosphamide, phosphoramide mustard, and doxorubicin are not able to cross the blood–brain barrier [59]. Rituximab has also poor CNS penetration because of its large size. Methotrexate (MTX) is currently the most effective single agent in PCNSL. Combining antimetabolites such as MTX and cytarabine (ara-C) constitute the backbone of most anti-PCNSL regimens with proven efficacy in prospective trials [60]. In a phase II study conducted by the International Extranodal Lymphoma Study Group (IELSG), 79 patients were assigned to 4 courses of MTX (3.5 g/m^2^) alone or combined with ara-C (4 doses of 2 g/m^2^), in both arms followed by whole-brain irradiation (WBRT). The addition of ara-C resulted in significantly improved response (CRR: 46 % vs 18 %; *p* = 0.006) and better outcome (3-year OS: 46 % vs 32 %; *p* = 0.07) compared with high-dose MTX alone, with manageable hematologic toxicity and uncommon nonhematologic side effects [61]. Another study highlighted the importance of ara-C dose, suggesting that 4 doses of 2 g/m^2^ is an appropriate choice [62]. Although it was not addressed in a phase 3 trial, the MTX-ara-C combination is currently the most commonly used treatment regimen outside clinical trials.

Methotrexate can also be administered as high-dosed monotherapy. The superiority of high-dose methotrexate over RT alone has never been demonstrated in a randomized trial [63]. Although not formally compared in a randomised trial, results of several studies suggest that the combination of high-dose methotrexate with RT is better than RT alone, in terms of increasing the proportion of long-term survivors (5-year survival 20–50 %) and OS by two to four times (median 30–72 months) [63]. There has been so far only one large prospective phase III study comparing consolidation WBRT to observation alone after high-dose methotrexate [64]. The unmet primary endpoint for non-inferiority and the high rate of protocol violations (only 318 out of 551 patients treated per protocol) prevent unfortunately any reliable conclusions to be drawn from this study [65].

In current practice, chemotherapy will usually be followed by consolidation RT (or high-dose chemotherapy) as initial treatment to maximize response and improve outcome; WBRT up to 24-36 Gy is recommended, without a boost, according to NCCN guidelines [6]. However, it has to be stressed that consolidation WBRT is associated with high rates of neurotoxicity, especially in patients > 60 years of age, and therefore often omitted. Several small retrospective series suggest that some elderly patients in CR after primary chemotherapy could be watchfully observed without OS impairment. To delay WBRT until relapse appears to be an acceptable strategy considering the increased risk of disabling neurotoxicity in these patients [60].

To reduce neurotoxicity, investigators explored reduced-dose WBRT (23.4 Gy in 13 fractions) for those patients achieving CR after induction chemotherapy with rituximab, methotrexate, procarbazine, and vincristine (R-MPV). Consolidation cytarabine was given after RT [66]. This phase II study was associated with a 2-year PFS rate of 77 %, median PFS of 7.7 years, and 3-year OS rate of 87 %. This R-MPV regimen with or without reduced-dose WBRT is currently tested by the Radiation Therapy Oncology Group (RTOG 1114 trial).

Building on the same induction immunochemotherapy (R-MPV regimen), consolidation WBRT can be replaced by a novel consolidation high-dose chemotherapy with thiotepa, busulfan and cyclophosphamide (TBC regimen) and autologous stem cell transplantation (HDC-ASCT). This strategy has been tested in a prospective, single-arm, phase II study (32 patients). Following R-MPV, objective response rate was 97 %, and 26 (81 %) patients proceeded with HDC-ASCT. There were 3 treatment-related deaths. Among all patients (N = 32), with a median follow-up of survivors of 45 months, 5-year overall survival (OS) and progression-free survival (PFS) were 81 % and 79 %, respectively. There was no evidence of neurotoxicity thus far. As stated by the authors, this intensive treatment should still be considered experimental. Moreover, the R-MPV regimen has not been formally tested alone, without a consolidation strategy such as WBRT or HDC-ASCT [67].

Outside clinical trials, outcome of CNS lymphoma remains globally dismal. About one third of patients with primary CNS lymphoma will present with disease that is refractory to first-line treatment. Even for patients achieving initial CR, about half of them will relapse. For refractory or recurrent disease, there are few treatment options available, and no established standard of care. For patients who previously achieved a long-lasting response to high-dose methotrextate, retained chemosensitivity might be assumed, and a rechallenge with methotrexate attempted. Conventional chemotherapy might be proposed as well. For selected patients, specific salvage therapies might be explored, including high-dose chemotherapy and ASCT [68], or stereotactic radiosurgery [69].

### New RT standards should translate into reduced toxicity

Historically, RT has been associated with significant risks of late toxicities and second malignancies in long-term survivors; relevant data are mostly derived from retrospective HL series [70–72].

The French-United Kingdom study of 4122 5-year survivors of various childhood cancers between 1942 and 1986 (median follow-up: 26 years) found that the average dose of radiation to the heart was linearly associated with the risk of cardiac mortality, with the incidence increasing by 60 % for every 1-Gy increase in mediastinal radiation dose [73, 74]. In a similar north-american cohort study restricted to female participants who had received chest irradiation for their childhood cancer diagnosis (RT treatment between 1970 and 1986), the cumulative incidence of breast cancer by age 50 years was 35 % among HL survivors [75]. At this point, it has to be stressed that these late-toxicity figures apply to patients treated for childhood cancers with ancient techniques, and cannot be directly extrapolated to mostly adult patients treated in these days with modern RT.

Different strategies have been implemented to reduce RT toxicities. As normal tissue complication rate is a function of dose and volume, RT therapeutic ratio should improve both with lower treatment doses and smaller target volumes. For NHL, there is now increasing evidence that tradional doses were higher than necessary for disease control; some investigators recommend now no more than 30 Gy for aggressive NHL or 24 Gy for indolent lymphomas [76].

Regarding target volumes for aggressive NHL, optimal imaging allowed to evolve from IFRT (encompassing the pre-chemotherapy involved node chains, based on anatomical landmarks) to involved site RT (ISRT) (target volumes reduced to cover the pre-chemotherapy involved nodes only) [3]. Such a strategy is based on the assumption that chemotherapy already eradicated adjacent or regional microscopic disease and ISRT targets the identifiable pre-chemotherapy disease. Clinical judgment in conjunction with the best available imaging is used to contour a clinical target volume (CTV) that will accommodate the uncertainties in defining the prechemotherapy gross tumor volume (GTV) in each individual case. In the situation where pre-chemotherapy imaging cannot be fused with the post-chemotherapy planning CT scan, allowances should be made for the uncertainty of the contouring and differences in positioning by including a larger volume in the CTV [3].

The proposed reduced late toxicity with involved-site or involved-node RT (INRT) is yet unproven; in the absence of recent long-term toxicity data, dose–volume metrics of organs at risk (OAR) will provide a surrogate measure of toxicity risk. In an Australian study comparing IFRT with INRT using conventional technique (parallel-opposed anterior and posterior photon beams), INRT allowed to reduce the proportion of tissue receiving higher RT doses (V95%) by a factor of 1.9. Regarding major organs at risk, the greatest benefit was seen when analyzing the dose received by 50 % of these organs (D50), which was reduced by half for the heart and the lungs. Keeping now the same INRT target volume and comparing between conventional technique and volumetic modulated arc therapy (VMAT), VMAT resulted in further relative reductions in cardiac dose, but without improvement in coronary artery dose-volume metrics, and at the expense of increased low-dose exposure to lungs [77].

A larger retrospective study (150 patients, 73 % with HL and 27 % with NHL) evaluated recently the risk of radiation pneumonitis (RP) after mediastinal RT with intensity modulated radiotherapy (IMRT) [78]. The overall incidence of any RP (RTOG grades 1-3) was 14 % among the entire group. However, patients who received salvage chemotherapy or transplantation for relapsed or refractory disease were at greater risk: the incidence of RP among those patients was 25 % versus 10 % among those who received consolidative RT only for newly diagnosed disease. In both groups, half patients developing pneumonitis required a steroid course (RTOG grade 3). Interestingly, disease bulk, history of smoking, pre-RT pulmonary function test values, and history of bleomycin toxicity did not predict RP. Regarding dosimetric factors, while the V20 (irradiated volume receiving ≥ 20 Gy) predicted RP risk, the volume of lung receiving lower doses of radiation, especially V5 (irradiated volume receiving ≥ 5 Gy), was the most powerful predictor of RP. If IMRT allows for a better conformality of the higher doses of radiation, these results are achieved at the costs of delivering a low-dose bath to large volumes of lung, leading obviously to a clinically meaningful lung injury. The risk of RP approaches 35 %, when >55 % of the total lung receives 5 Gy in the treatment of HL or NHL with IMRT.

Different IMRT and VMAT refinements have been recently described, to optimize dose delivery and better spare OAR. Traditional IMRT beam arrangements involving 9 fixed and equally distributed beams produce a low-dose bath to the all surrounding critical organs; RT planning should therefore not be automated, and beam angles should be carefully selected on an individual basis, leading for example to a 5-beam anterior-posterior weighted “butterfly” coplanar beam arrangement [79]. A single arc VMAT plan can also be replaced with a more sophisticated three-arcs VMAT (“butterfly” VMAT), with two anterior and posterior coplanar arcs of 60°, complemented with 1 no-coplanar arc of 60° [80].

Another possibility to further reduce the target volume and thus the irradiated volume is the use of gating techniques such as deep-inspiration breath-hold. Combined with IMRT, it can greatly reduce radiation exposure of the coronary arteries, heart, and lungs in patients with mediastinal HL [81]. Despite the obvious advantage to reduce side effects related to the heart and the coronary arteries, an open question is the impact of intensity modulation techniques on the incidence of second malignancies. While the application of VMAT techniques can result in better dose spearing of the thyroid gland, the heart and the coronary ostia, deep-inspiration VMAT was actually shown to be inferior to classical parallel opposed treatment techniques with regard to second cancer induction; VMAT can increase for example the cumulative absolute risk of breast cancer induction by 100 %. VMAT should therefore be cautiously implemented in clinical practice [82].

Last, proton therapy may offer significant and clinically relevant advantages to spare important OAR’s, and has been endorsed by NCCN as an appropriate treatment modality for NHL [6]. While several published studies have evaluated the use of proton therapy for HL, data regarding patients treated with proton therapy for NHL are scarce. Early outcomes appear favorable, but longer follow-up is needed before drawing any definitive conclusions [83].

## Conclusions

NHLs exhibit both high chemosensitivity and radiosensitivity. The excellent response rates achieved over the last 15 years with R-CHOP treatments in aggressive lymphomas, and with rituximab alone or in combination with chemotherapy in indolent lympomas have lead to the premise that RT might be obsolete, causing unnecessary toxicities without impacting favorably on outcome. Two large observational studies in FL and DLBCL demonstrate a clear trend towards RT-free regimens in the most recent years. However, abandonment of RT in favor of (chemo-) immunotherapy alone might negatively affect patient OS. Omitting RT in the treatment of NHLs should not be considered outside of well conducted prospective studies.
